# Place Effects and Chronic Disease Rates in a Rural State: Evidence from a Triangulation of Methods

**DOI:** 10.3390/ijerph17186676

**Published:** 2020-09-14

**Authors:** Mohamed Shabani Kariburyo, Lauri Andress, Alan Collins, Paul Kinder

**Affiliations:** 1 Division of Resource Economics and Management West Virginia University, Morgantown, WV 26506-6108, USA; Alan.Collins@mail.wvu.edu (A.C.); Paul.Kinder@mail.wvu.edu (P.K.); 2 Health Policy, Management, and Leadership, School of Public Health West Virginia University, Morgantown, WV 26506-9190, USA; LAANDRESS@hsc.wvu.edu

**Keywords:** food environment, food insecurity, chronic diseases, rural food access, spatial analysis, matching methods, D12, I12, I14, L81

## Abstract

High rates of chronic diseases and increasing nutritional polarization between different income groups in the United States are issues of concern to policymakers and public health officials. Spatial differences in access to food are mainly blamed as the cause for these nutritional inequalities. This study first detected hot and cold spots of food providers in West Virginia and then used those places in a quasi-experimental method (entropy balancing) to study the effects of those places on diabetes and obesity rates. We found that although hot spots have lower rates of chronic diseases than non-hot spots and cold spots have higher rates of chronic diseases than non-cold spots—the situation is complicated. With the findings of income induced chronic disease rates in urban areas, where most hot spots are located, there is evidence of another case for "food swamps." However, in cold spots which are located mainly in rural areas, higher rates of chronic diseases are attributed to a combination of access to food providers along with lacking the means (i.e., income for low-income households) to form healthier habits.

## 1. Introduction

Poor diet is a modifiable risk factor for obesity and diabetes. Contributing to substandard nutrition, the food environment is also thought to be a primary driver of poor diet. Health advocates, community leaders, and researchers are worried that food environment problems, and poor diets in general, may be more severe in certain low income and rural American communities because these areas have limited access to affordable and nutritious foods. A primary concern is that some poor or rural areas do not have access to supermarkets, grocery stores, or other food retailers that offer the large variety of foods needed for a healthy diet (for example, fresh fruits and vegetables, whole grains, fresh dairy, and meat products) [1,2,3,4]. Instead, individuals in these areas may be more reliant on food retailers or fast food restaurants that only offer more limited varieties of foods. It is hypothesized that the relative lack of access to full-service grocery stores and the easier access to fast and convenience foods may be linked to poor diets, and ultimately, to obesity and other diet-related diseases [5,6].

It was this concern that led Congress, in the Food, Conservation, and Energy Act of 2008, (hereafter referred to as the 2008 Farm Bill) to instruct the U.S. Department of Agriculture (USDA) to conduct a 1-year study assessing the extent of the problem of "food deserts." More specifically, the remit in the 2008 Farm Bill called upon the USDA to study the limited access to food, characteristics, and causes of limited access, the effects limited access has on local populations and recommendations for addressing the causes and effects of limited access. As a result, numerous researchers have tried to empirically measure the association between the food environment and obesity and diabetes during this past decade. These studies have investigated whether living in a “food desert” or “food swamp” caused higher rates of these chronic diseases or increased nutritional inequality between the wealthier and the less fortunate population [7,8,9]. Food deserts are often described as residential areas with limited vendors of affordable and healthy foods [8]. In comparison, food swamps are areas with a higher number of unhealthy vendors, such as convenience stores and fast food restaurants [10]. Despite this surge of researchers, most studies still failed to prove the causal claim that underserved areas are associated with an increased risk of chronic diseases, with few exceptions [8,10]. Nonetheless, federal and local governments continue to support the supply-side through initiatives such as the Healthy Food Financing Initiative and the Agricultural Act of 2014. (Since 2011, the healthy food financing initiative has been providing subsidies to grocery stores, farmers markets, and other actors working in underserved areas. The Agricultural Act of 2014 allocated about 125 millions in federal funds to support access to healthy food in underserved areas [9].)

It is in this context that some researchers have started to question the metaphor of a "food desert" and its applicability in different geographical entities in the United States. This argument states that studies in this field should not just measure the food environment as binary—areas where people live in a food deserts or food swamps, and areas where people do not live in these regions. Instead, it is important to consider how locals perceive their food environment [2,11]. This is a way to include the spatially-explicit factors that individuals have to face on a daily basis. Furthermore, the food environment is believed to be different in urban and rural areas—what is considered a "food swamp" in one area might be a "food oasis" in another [12]. Therefore, to understand the impact of the food environment on chronic diseases, it is crucial to properly define the food environment spatial measure.

In this research, we explicitly account for spatial drivers that may influence the supply of food vendors and study how these places might impact diabetes and obesity rates in West Virginia. This state is an interesting case to study. It leads the nation in obesity rates and is ranked the second-highest in diabetes rates nationally (based on 2018 statistics provided by the West Virginia Department of Health and Human Resources Bureau for Public Health (https://dhhr.wv.gov/hpcd/data_reports/pages/fast-facts.aspx)). This study will add to efforts that evaluate and understand how the neighborhood retail food environment contributes to these chronic diseases. In economics and public health literature, this paper is related to articles that studied the causal link between the food environment and chronic diseases ([8,9,10,11,13,14,15]). We combine the hot spot analysis results with a quasi-experimental approach (entropy balancing) to study how diabetes and obesity rates might be impacted by these places (hot and cold spots). To our knowledge, this is the first study to combine results from the hot spot analysis and a quasi-experimental approach to address this issue. First, we wanted to test whether places with an abundance of food providers (hot spots) and those that lack food providers (cold spots) are associated differently with diet-related diseases. Second, we wanted to understand whether the difference in these chronic diseases rates between hot (and cold) spots depends on whether a census tract is urban or rural. Finally, we wanted to test whether there is a significant diet-related disease–income relationship between hot (and cold) spots.

The remainder of the article is organized as follows: in Section 2, a literature review on chronic diseases and place-based policies is elaborated; Section 3 explains the methods and the data used in this study; Section 4 presents the results; Section 5 provides some discussion and policy implications; finally, Section 6 discusses about the limitations of this research.

## 2. Literature Review

Studies associating chronic diseases and the overall food environment across different geographical regions have been surging in economics and public health fields. These studies consider whether neighborhood food vendors play a role in nutritional inequality, and therefore, chronic diseases.

The literature behind the associations between healthy and unhealthy food outlets and chronic diseases has heavily relied on spatial analysis or geographic information system data ([5,9,14]). To examine the association between food deserts (and food swamps) and consumption of snacks/desserts or fruits/vegetables, Hager et al. combined geocoded home addresses and maps of food deserts/food swamps [16]. They found that adolescent girls living in an unhealthy environment (food swamps) had a higher intake of snacks/desserts than girls not living in these areas. Further tackling the issue of food swamps and food deserts, Cooksey-Stowers et al. combined commercial street reference, socioeconomic, food environment, and obesity datasets [8]. Their results suggest that food swamps are associated with higher obesity rates, but the absence of a full grocery store shows inconclusive results. Jang et al. investigated the intersectionality of race/ethnicity and poverty in terms of spatial access to supermarkets, grocery stores, and convenience stores in the Detroit metropolitan area [17]. They suggest that place-based policies can successfully improve health intervention strategies when implemented by federal, state, and local authorities.

By further examining nutritional inequalities in food swamps and food deserts, Phillips et al. found that the association between hospitalization rates among adults and diabetes followed a curvilinear relationship, plateauing at the saturation point of unhealthy food stores [10]. Using a measure of diabetes incidence, Christine et al. showed that having resources that support physical activity and healthy diets in the neighborhood can lower the incidence of type 2 diabetes [18]. In most research, the definition of chronic disease measures is clearly specified with the association between food environment and diabetes incidence [18,19] being analyzed, and the relationship between food swamps and a measure of severity being treated in [10].

There are also recent articles, using large, nationally representative samples that have started to question the hypothesis that the neighborhood food environment (i.e., supply side factor) reduces nutrition inequality. Allcott et al. argued that increasing the supply of healthy groceries alone does not reduce nutritional disparities ([9,20]). Using counterfactual simulations, they demonstrated that simply fixing food deserts by adding stores or responding only to supply-side issues achieves merely a ten percent reduction in nutritional inequality. Alternatively, the researchers assert that the remaining ninety percent difference in nutritional inequality might be driven by any number of demand-side factors in their simulations, including education, prices, and/or preferences [9].

Johnson et al. highlighted a vital trend in the neighborhood food environment literature—most research on this subject tends to be on urban communities and rural communities are mainly left behind [21]. Bailey sounded the alarm on how traditional grocery stores are becoming scarce in rural America [22]. Using a cross-sectional study, Sharkey showed that in Texas border colonia households, convenience stores were the critical variable that influenced nutrients intakes [12]. The demand for food in rural America relies on many different healthy and unhealthy stores due to the shortages in the supply. Some households depend on dollar stores, big box retailers (e.g., Walmart), convenience stores (e.g., Sheetz), fast food restaurants, and farmers markets [12,21]. Understanding places might be the missing point in solving nutritional and health disparities in these communities. Johnson et al. focused on rural areas and identified determinants related to access to healthy and affordable food to improve population health and reduce health inequalities in rural America [21].

Further, studies focusing on either rural or urban areas may differ in findings because of the study area and empirical methodologies. Alviola et al. found that fast-food restaurants and convenience stores were the main determinants of food deserts in a rural state like Arkansas [15]. Allcott et al. demonstrated how the addition of a healthy store in certain geographical areas does little in reducing nutritional inequalities. Their national representative data helped them advance some policy implications that could help federal, state, and city officials in their intervention strategies [9].

Overall, much of the previous research has highlighted the need to provide more evidence on the effect of supply inequities on chronic diseases. The lack of causal effect studies that try to understand the association between chronic diseases and food environment leaves many unanswered questions on this matter. By evaluating how places in a largely rural state impact diabetes and obesity rates, our research aims to pinpoint areas that are at risk of chronic diseases.

## 3. Data and Methods

To understand the relationship between West Virginia’s food environment (place effects) and chronic diseases, this research focuses on food provider geographic locations with the caveat that while other factors impacting food access certainly exist, they are beyond the scope of this research. Our specific approach to this issue offers a triangulation of methods where we combine results from a spatial analysis method (hot spot analysis) with a matching technique (entropy balancing), and an ordinary least square (OLS) regression to understand whether the extent of food providers’ presence and/or absence impacts chronic diseases rates in West Virginia. Recent literature reviews suggest that this combination of methodologies to identify the effects of retail food providers on chronic disease rate incidence constitutes a new approach in this literature [23]. (Reference [23] utilized the same logic while studying the economic impact of organic agriculture hot spots in the United States. The main methodological difference between the two studies is that [23] computed the hot spot analysis then used the propensity score matching (PSM), whereas our study used the entropy balancing method and perform a sensitivity analysis using the propensity score weighting and the kernel matching method.)

### 3.1. Hot and Cold Spots (Place Effects)

To capture the geographic distribution and patterns of food stores in West Virginia, this research utilizes a dataset from the WV Foodlink project (http://foodlink.wvu.edu/) housed at the food justice laboratory within the Department of Geology and Geography at West Virginia University. In collaboration with their community partners, this laboratory has developed a resource hub and learning commons to support a people-centered, resilient food network in West Virginia. These data contain the geolocation of charitable assistance agencies, big box retailers, grocery stores, small box retailers, convenience stores, farmers markets, and undefined SNAP accepting retailers. In this data, we count 68 big box stores, 996 convenience stores, 134 farmers markets, 320 grocery stores, and 587 small box retailers. Toward the end of 2015, the WV Foodlink project verified the validity of these locations by sending participants from this project to the specific site of a food store (or outlet) to confirm their actual latitude and longitude. In this research, food stores geographic coordinates were used as a basis in all spatial analysis conducted.

In the spatial analysis practice, researchers usually use two methods to map clusters: heat maps and hot spot analysis. The heat map as illustrated in Figure A2 utilises a color gradient that indicates areas of increasingly higher density. In our map, food vendors tend to cluster around West Virginia larger cities—and it is very difficult to visualize where the population is lacking food vendors—which is why we focus on the hot spot analysis in this research. Hot spot analysis is a statistical method that assesses geographic clustering. This method is a local indicator of spatial autocorrelations (LISAs)—it uses variations in local spatial autocorrelation compared to surrounding areas, or areas in which clusters of density or intensity are statistically distinct from the neighboring landscape—an option that a heat map does not offer.

**Figure A1:**
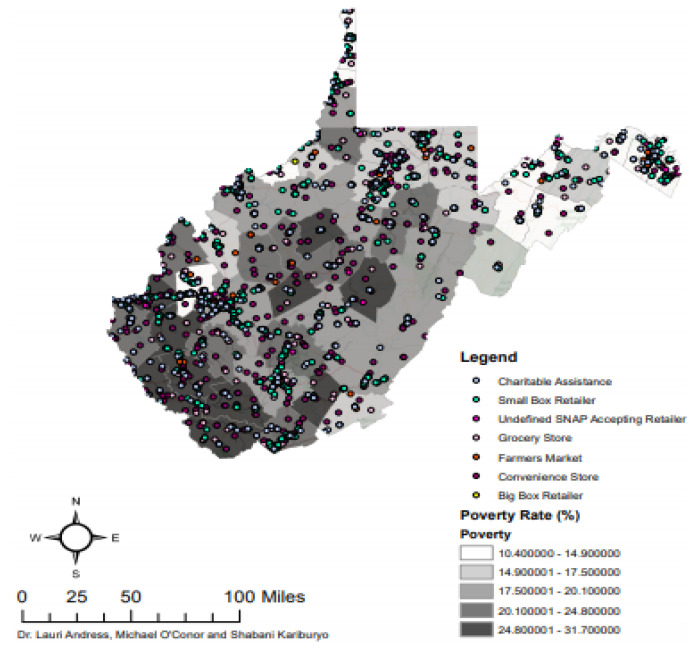
Food retail distribution and poverty rates.

**Figure A2:**
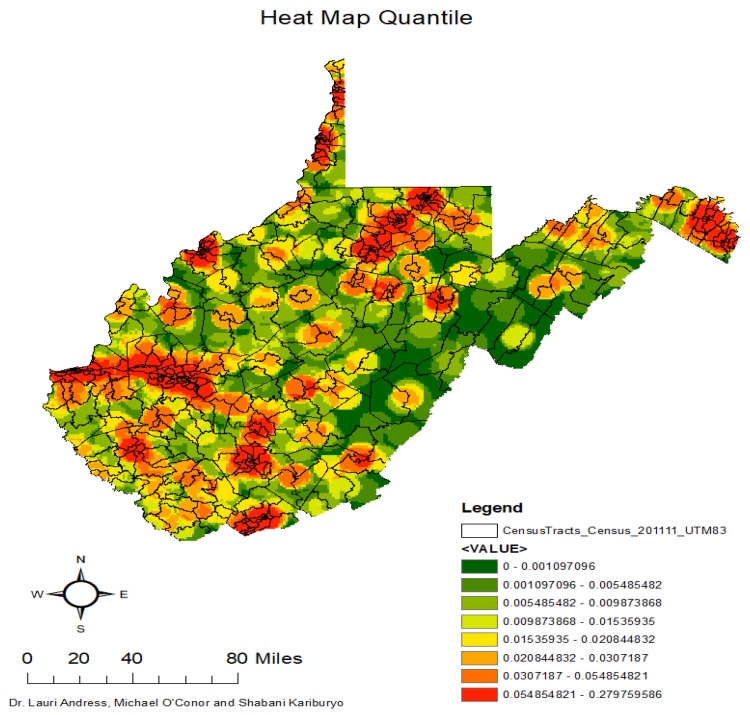
Heat map of food providers in West Virginia.

Figure A1 in the appendix displays the specific latitudes and longitudes of all the stores in West Virginia. Each point, as described in the legend, represents a specific type of store. Separating between healthy and unhealthy food suppliers is one option that this research could have followed; however, in some areas in West Virginia, families rely on stores that are considered unhealthy to purchase all their fresh produce. (According to the American Healthy Food Financing, West Virginia is facing a growing problem of store closures—leaving behind thousands of families. As a result, families rely more and more on closer stores such as convenience stores or Mom and Pop stores; see https://www.investinginfood.com/commentary-w-v-projects-try-new-ways-to-deliver-fresh-food-options/.) To account for these inequities, this research defines a hot spot as an area which has a much higher than average count of food providers, and a cold spot as an area which has a much lower than average count of food providers. (Clusters of stores whose values are atypically high or low compared to the entire sample. Given a chosen level of statistical significance (e.g., *p* < 0.001), the hypothesis test identifies high (low) value observations surrounded by other high (low) value observations, where the difference between values observed for these identified clusters and those for the surrounding observations is too great to be the result of random chance. For example, Kanawha county, which is in a hot spot, has 30 grocery stores, 5 big box stores, 80 small box stores, 96 convenience stores, and 1 farmers market. Pocahontas county, a cold spot in our analysis, has 7 grocery stores, 0 big box store, 4 small box stores, 1 convenience store, and 0 farmers markets.) Optimized hot spot analysis (for more details on this method please see the ArcMap manual: https://desktop.arcgis.com) is used as a spatial data mining tool that asks the data (in our case, latitude and longitude of each specific store) to obtain the settings that yield the optimal hot spot results. It uses the Getis-Ord $G_{i}$* which works as follows:

(1) $$G_{i}=\frac{\sum_{j=1}^{n}w_{i,j}x_{j}-X^{bar}\sum_{j=1}^{n}w_{i,j}}{S\sqrt{\frac{n\sum_{j=1}^{n}w_{i,j}^{2}-{\left(\sum_{j=1}^{n}w_{i,j}\right)}^{2}}{n-1}}}$$

where $x_{j}$ is the attribute value for food provider *j*; $w_{i,j}$ is the spatial weight between food providers *i* and *j*. The choice of a weight matrix is a very important step during the hot spot analysis and in our case, we use an inverse distance weight matrix. This distance-based weight matrix with a distance band of 10 miles between food providers because the USDA defines low food access census tracts as those who have 500 persons and/or at least 33 percent of the population who live more than 10 miles from a supermarket or a large grocery store (https://www.ers.usda.gov/data-products/food-access-research-atlas/documentation/). Using this type of distance-based weight matrix, a food provider that is farther away, receives less weight in the analysis. *n* is equal to the total number of features and $X^{bar}$ is just an average of *n* attribute values across West Virginia. Lastly, Equation (2) is the square root of the average of the squares of the sum of all attributes minus the average attribute squared.

(2) $$S=\sqrt{\left(\frac{\sum_{j=1}^{n}x_{j}^{2}}{n}\right)-{\left(X^{bar}\right)}^{2}}$$

The $G_{i}^{*}$ statistic can be thought of as a z-score so that no further statistical calculation is required.

**Figure 1:**
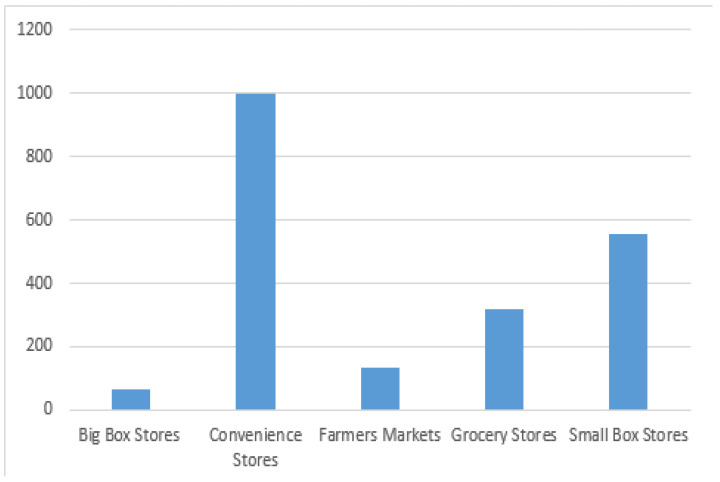
Food retail distribution in West Virginia.

**Figure 2:**
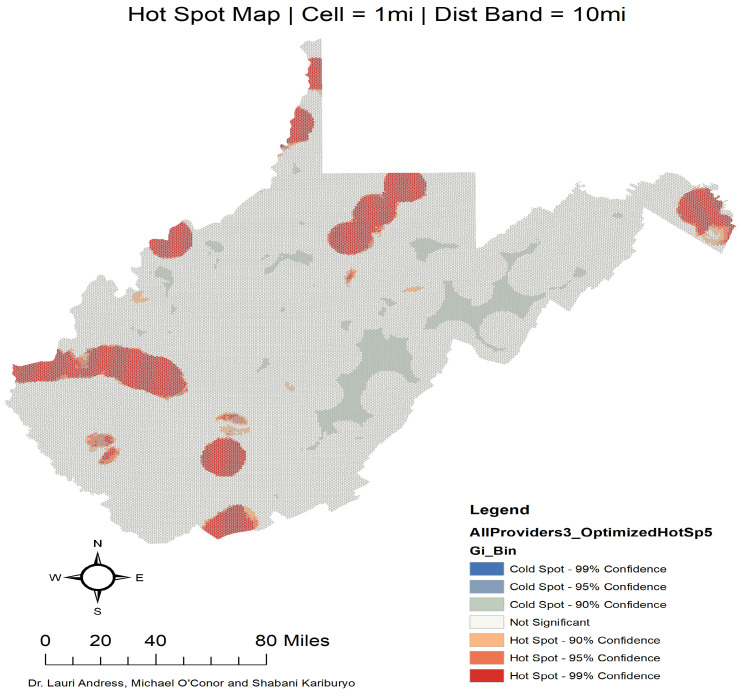
Hot spots and Cold spots of food providers in West Virginia.

Figure 1 clearly shows the highly uneven food environment landscape in West Virginia. The most common stores in West Virginia are convenience and small box retailers which make up about 76 percent of the retailers in the state. This unevenness was the reason why it was important to row standardize this matrix to give each feature the same weight in the analysis. Figure 2 shows a map depicting the results of the hot spot analysis. Among the 484 census tracts in West Virginia, those red areas show census tracts that are hot spots of food providers at a 99 percent confidence interval. The light gray areas are census tracts that represent cold spots at a 90 percent confidence interval. Every statistically significant hot or cold spot area was reported (Figure 2 displays a legend with the level of statistical significance at the 90, 95, and 99 percent; we used these visual explanations to designate a census tract as hot or cold spots).

Using the results from this map, a census tract level indicator variable is created, which takes a value of 1 if the census tract is identified as being part of a statistically significant hot or cold spot, and 0 otherwise. To examine the impact of hot and cold spots on chronic diseases, this research characterizes hot and cold spots as being “treatments”. It measures the impact of the treatment on a census tract diabetes and obesity rates.

### 3.2. Entropy Balancing

A big challenge of this empirical work is to establish an econometric link between the chronic diseases of diabetes and obesity and those places identified as hot or cold spots for food providers. Several endogenous variables might lead a census tract to being identified as a hot or cold spot. To mitigate the potential endogeneity issue that might arise with being in a hot spot or cold spot, we employ a matching technique.

Since the analysis is based on the concept that a hot or cold spot represents a treatment—our measure of interest is the average treatment effect on the treated ATT, which is defined as:

(3) $$ATT=E\left[y_{1}/D=1\right]-E\left[y_{0}/D=1\right]$$

where *D*, is an indicator variable that is equal to 1 if a census tract is a hot (or cold) spot and 0 otherwise. For a specific census tract, we denote $E[y_{0}/D=1]$ as the counterfactual—that is, the outcome of a census tract in a hot (or cold) spot would have been realized had they not been positioned in those geographical locations; and $E[y_{1}/D=1]$ as the outcome after being treated as a hot (or cold) spot. The counterfactual is unobservable in this case; therefore, a suitable proxy to solve for ATT is needed. In the case of a randomized control trial, the average outcome of units not exposed to treatment, $E[y_{0}/D=0]$, is a proper substitute. However, as discussed before, being in a hot or cold spot, and thus, selection into treatment, could be endogenous [24,25].

The matching process’s key idea is to facilitate a situation of randomization where the assignment to the treatment can happen by a chance procedure. The unobserved counterfactual outcome is assigned by matching the treated units with untreated units that are as similar as possible with regard to all pretreatment covariates that are associated with selection into treatment (being in a hot or cold spot) and impact the dependent of variables of interest (diabetes or obesity rates). The estimate representing ATT based on matching can be written as follows:

(4) $$ATT\left(x\right)=E\left[y_{1}/D=1,X=x\right]-E\left[y_{0}/D=0,X=x\right]$$

*x* is a vector of relevant covariates, which are described in the following subsection, $E[y_{1}/D=1,X=x]$ is the expected outcome for the census tracts that were in the hot (or cold) spots, and $E[y_{0}/D=0,X=x]$ is the expected outcome for the best matches of the hot (or cold) spots census tracts [26].

Studies that have investigated the effectiveness of place-based policies have used nearest neighbor matching, propensity score matching, Mahalanobis distance matching, and entropy balancing [27,28]. Marcus et al. argued that because of the sensitivity to the choice of matching variables, entropy balancing balances the matching covariates more effectively than the propensity score matching [24]. Furthermore, Deb et al. elaborated a summary of the main difference between entropy balancing and the propensity score matching: the former does not require the researcher to first estimate the weights, and then verify whether the treatment and the control covariates balance like the latter does [29]. Hainmueller showed that entropy balancing is better suited than either the propensity score or Mahalanobis distance matching. Entropy balancing generates a weighting scheme that reweights control observations so that they are perfectly balanced compared to treated individuals based on the mean moments of covariate distribution. After weighting, the treated indicator is orthogonal to covariate moments included in the entropy balancing exercise. Finally, the weights are then incorporated in Equation (5) as a survey weight (one can think about these survey weights or weights as similar to those that are created by the logit model of the propensity score analysis [25]):

(5) $$Y_{i}=\beta_{0}+x_{i}\beta +\epsilon_{i}$$

where $Y_{i}$ is the rate of diabetes or obesity; and $x_{i}$ is a set of independent variables that includes whether a census tract is considered in treatment (hot spots and cold spots), an urban versus rural designation for each census tract, mean household income, and the proportions of the population that are employed in the sectors of agriculture, fisheries, hunting, and mining (AFHM). The AFHM variable was selected because it is important to understand the relationship between these sectors, which have been hit harshly by economic changes, and the food access inequality [30]. Finally, Mactaggart investigated how health disparities in rural settings may be exacerbated by AFHM occupations [31].

The independent variables of interest include those to test the first set of key hypotheses that diabetes and obesity rates differ in hot (and cold) spots compared to non-hot (and non-cold) spots. Other hypotheses are examined with interaction variables between the treatments and urban, rural, and household income variables. These hypotheses state that urban or rural aspects of census tracts accentuate the treatment differences; and there exist different chronic disease–income relationships at various income levels and across treatment and non-treatment census tracts.

### 3.3. Data

Our triangulation of methods employs three publicly available datasets: food store locations (Source: WVfoodlink project), socioeconomic variables (Source: American Community Survey (ACS)), and health outcomes (Source: County Health Rankings and Roadmaps). Given difficulties in finding experimental and observational data for diabetes and obesity at the census tract level, a metric was developed through the following steps. The County Health Rankings and Roadmaps provide the data for the percentage of adults aged 20 and above with diagnosed diabetes at the county level. The same source also provides the percentage of adult population (age 20 and older) that reports a body mass index greater than or equal to 30 kg/m^2^ which is considered as obese. In this research, the percentages of each county’s diabetes and obesity were distributed to each census tracts.

Using previous research on the determinants of neighborhood food environments [14,15], the following socioeconomic characteristics were used as covariates to control for the selection into the treatments: number of family households; individuals who were age 25 and above who had a bachelor’s degree; estimated vehicles available; households that received food stamp/SNAP benefits in the pasts 12 months; mean household income (adjusted in 2016 dollars); unemployed civilians who are in the labor force; age groups (<19 and >59); race (the proportion of non-white population); and health insurance coverage. Finally, community health centers (CHS) in each census tracts served as a control for health services. These data originated from the West Virginia Primary Care Association.

## 4. Results

### 4.1. Descriptive Statistics

Table A1 shows the sample means of all matching covariates among four groups: (1) hot spots as the treatment group when a census tract is in a hot spot, (2) non-hot spots that are the control group for hot spots, (3) cold spots as the treatment for cold spot analysis, and (4) non-cold spot control census tracts. Note that there are difference columns between the groups along with the corresponding t-test statistics with a null hypothesis of difference equal to zero.

Table A2 compares the means of all matching covariates across the treatment groups and the synthetic group (control) obtained via entropy balancing. This table illustrates how entropy balancing improves the quality of matching between hot (cold) and non-hot (non-cold). When we compare the realizations of the pre-treatment characteristics of the hot spots and cold spots to those of the control group, the results reveal the efficacy of entropy balancing. After matching, all covariates are perfectly balanced, and no difference remains. Therefore, the control group in the next regression analysis is comprised of credible counterfactual for the sample of census tract that are hot or cold spots.

### 4.2. Results

The appendix presents baseline model results to assess the impacts on diabetes and obesity rates from solely the treatment variable of populations residing in either a hot or a cold spot (Table A3 and Table A4). From these models, results consistently show that hot spots are healthier environments with decreases in diabetes and obesity rates compared to non-hot spots, while cold spots increase the rates for both chronic diseases compared to non-cold spots. In addition, both unbalanced and balanced models are estimated to compare the unbalanced and the entropy balanced models. As is conventional in the literature [25], these results illustrate the overestimated bias observed from the unbalanced models compared to the corrected balanced models. Our interpretation is that by not accounting for covariates, such as the number of vehicles in a census tract, the mean household income, education, and age groups, this absence could have inflated the effect of living in non-treatment census tracts on diabetes and obesity rates.

Our main results consist of eight regression models showing four sets of treatment effects for hot and cold spots, respectively (Table 1 and Table 2). These models examine the impacts of interactions between urban and rural designation with hot spots (columns (1) and (2)) along with the same interaction impacts with cold spots (columns (3) and (4)) on diabetes (Table 1) and obesity (Table 2) rates. For urban hot spots, both diabetes (β = −0.0084, *p*-value = 0.095) and obesity (β=−0.0278, *p*-value = 0.004) rates are lower than non-hot, urban, and all rural census tracts. The reduction in obesity rates is particularly notable as it reflects a decrease of seven percent from the statewide average of 37.7 percent in 2016. Urban cold spots had mixed impacts on chronic diseases with a small, statistically significant impact on diabetes (β = −0.0061 *p*-value = 0.089) and a zero statistical impact on obesity rates. For rural census tracts, the treatment of either a hot or cold spot increased both diabetes and obesity rates by 1–3 percentage points relative to urban census tracts and rural, non-hot, or non-cold spots.

In Table 1 and Table 2, we also sought to determine whether diabetes and obesity rates in hot (and cold) spots are impacted differentially by differing household income levels. Within urban areas, the results point out that as income increases for the top household income quartile (75 percent), both diabetes and obesity rates increase within hot and cold spots. Since, as mentioned earlier in this manuscript, the food landscape in West Virginia is driven by convenience stores and small box retailers, these increases in rates could be evidence of food swamps causing chronic diseases ([8,10]).

In urban cold spots, we still found that there is no evidence of a diabetes rate differential among the lower and middle household income quartiles, and their counterparts in urban non-cold spots. However, we found that an increase in earnings of the top household income increases the rate of diabetes in urban cold spots. This result reinforces the view that urban areas are food swamps in West Virginia. In rural census tracts, the treatment-income interaction effect occurs primarily at the 50 percent and lower household income quartiles with statistically significant, negative impacts on chronic disease rates. The largest impacts from the treatment-income effect are on obesity rates. As an example, a one percent change in household income for the population at the 50 percent quartile in rural hot spots results in a 0.0003 percent reduction in obesity rates. When compared to the rural hot spot treatment increase in obesity rates of 0.0185 percentage points, household income at the 50 percent quartile would have to rise over 60 percent to offset the treatment effect of a hot spot in a rural area.

### 4.3. Sensitivity Analysis

Our sensitivity analysis is decomposed into two parts: first, we relied on the propensity score weighting and the kernel matching method to analyze the sensitivity of our results—using other matching procedures gave us a sense of reassurance in the plausibility of our results. As explained earlier, these two methods are different to the entropy balancing approach because the propensity score weighting assigns to each control a weight that is equal to (1/(1−P(X)) where P(X) is the propensity score; the kernel matching method uses the observed propensity scores to match treatment and control units while using the closest covariates in terms of propensity scores.

Second, we used the data from year 2017 (t+1) to verify whether our conclusion holds. Since the locations of all food vendors were verified in 2016, we also ran the entropy balancing model for the year of 2017 because most of the covariates used in the matching process were collected from the American Community Survey (ACS) 2014-2018. Thus, this process helps us understand how our estimates change when we look at another year (t+1) that is close to the time period for which we have data on the geolocations of food providers in West Virginia.

There are several similarities between our main model, entropy balancing (2016), and the propensity score weighting, the kernel matching method, and the 2017 results. Using the same logic, i.e., starting with regressions that include the interactions between the different treatments (urban and rural hot (and cold) spots) and the different income quartiles, we still find the same results for both techniques. However, the coefficients differ in magnitude with the entropy balancing method. The results are econometrically encouraging per se—the combination of the hot spot analysis and the entropy balancing is a solid way of understanding how the food environment affects chronic diseases in certain geographical entities; i.e., these results are not sensitive to time.

Overall, Table A5, Table A6, Table A7, Table A8, Table A9 and Table A10, the results of our sensitivity analyses, inform us that obesity and diabetes models hold up well. When comparing the range of treatment coefficient estimates for entropy balancing (2016) to the propensity score weighting, the kernel matching, and the entropy balancing for the year 2017 for hot and cold spots—the signs of almost all the coefficients are the same as in our entropy balancing model (2016). The only difference resides in the magnitudes of the coefficients. However, the entropy balancing results from the 2017 data are almost the same as those from our original models.

## 5. Discussion

Our findings are of relevance to policymakers in rural states such as West Virginia. Overall, these results highlight the fact that places in West Virginia with access to different food vendors (hot spots) tend to have chronic disease outcomes associated with food swamps. This finding is not new in the literature. However, most researchers tend to separate between healthy and unhealthy food stores and find that individuals who have access to stores that offer a variety of food are healthier than inhabitants of food deserts [32]. In this research, we found that explicitly accounting for spatial forces (hot and cold spots) is better suited for a region where a food oasis might be the only choice. In a rural state such as West Virginia, some areas depend on convenience stores or Mom and Pop shops for all their groceries (https://www.investinginfood.com/commentary-w-v-projects-try-new-ways-to-deliver-fresh-food-options/). Figure 2 shows that large numbers of cold spots are in the eastern part of West Virginia where Randolph, Pocahontas, Webster, and Tucker counties are considered mountainous and largely rural.

Therefore, separating between healthy or unhealthy stores might not do justice to residents of cold spots for example. Investigating whether a store is in an urban or rural area seems to tease out better the chronic disease–income relationship of individuals in that area. Moreover, our results also inform us that socioeconomic status (SES) plays a primal role, whether you live in a cold spot or a hot spot. On the one hand, these results showed consistent evidence that income growth in the top income quartile increases both chronic diseases within urban hot and cold spots. On the other hand, household income increases among the 50 percent quartile and lower income groups are linked to lower rates of chronic diseases within rural census tracts.

This research examined the differential impacts of income and access to retail food providers in West Virginia. After controlling for socioeconomic variables in hot and cold spots, we found that urban and rural areas in West Virginia are affected differently by the food landscape. First, we found that the household income impacts on chronic diseases among the top earners in both hot and cold spots located in urban areas are essentially zero. Thus, policies that aim to reduce chronic disease rates by improving access to healthy food alone in urban areas of West Virginia might not work as the problem could be more related to diet preferences than food access. As an explanation, we note that more than 76 percent of the food retail stores in West Virginia are composed of convenience stores and small box retailers so that households are more likely to patronize these types of stores. Phillips et al. highlighted the issue of food swamps and diabetes-related hospitalization and found a positive relationship between diabetes-related hospitalizations and a higher index of food swamp severity [10].

Second, we consistently found that rural cold spots have higher chronic disease rates, and at the same time, increasing household income for the lower-income groups in these cold spots results in a lowering of chronic disease rates. Thus, improving access to healthy food providers might work in rural cold spots. Therefore, policies such as the healthy food financing that offer food retailers subsidies to locate in underserved areas should be encouraged in rural cold spots. Nonetheless, these policies should be accompanied by some means-tested benefits in order to boost the demand for nutritious food by low-income households. Such a policy recommendation is supported by other researchers who currently lobby for increases in SNAP benefits ([9,20]). Our findings that income increases among low-income households reduce rates of chronic diseases shows that given the opportunity, these households would improve their healthy habits.

## 6. Limitations

Despite significant contributions in methodology and implications, a number of limitations should be acknowledged. First, when examining the food environment in West Virginia, we did not separate between healthy stores and non-healthy stores. Figure 2 in our study demonstrates how stores that sell food in West Virginia are largely dominated by convenience stores. Without a detailed datasets on how people shop at the census tract level, it is almost impossible to capture the effect of a healthy store on the health conditions of different demographics in a rural state such as West Virginia. This issue also did not allow us to study whether the differences in obesity and diabetes rates between hot and cold spots and their comparable non-hot and non-cold spots census tracts are driven by demand tastes or by the supply (as we suggest in this study). Second, future studies should utilize spatially measured data for diabetes and obesity rates collected at the census tract level. Finally, the Center for Disease Control (CDC) includes individuals who have diabetes and severe obesity within the group of those who are at a higher risk of being severely affected by COVID-19 (https://www.cdc.gov/coronavirus/2019-ncov/need-extra-precautions/groups-at-higher-risk.html#severe-obesity). Due to the lack of data for the years 2016 and 2017 and given this recent global health issue, this research did not include this variable—a new avenue of research for further studies would be to understand how place effects that impact food intakes are associated with this novel pandemic.

## Figures and Tables

**Table 1:** The impacts of urban and rural hot (and cold) spots on diabetes (2016).

Diabetes	(1)	(2)	(3)	(4)
Treatment: Urban Hot Spots	−0.0084 * (0.095)			
Treatment: Rural Hot Spots		0.0156 *** (0.005)		
Treatment: Urban Cold Spots			−0.0061 * (0.089)	
Treatment: Rural Cold Spots				0.01246 *** (0.001)
Ln (Income) 25th percentile	−0.0024 (0.629)	0.0020 (0.581)	−0.0087 *** (0.001)	−0.0042 * (0.061)
25th percentile * Treatment	0.0043 (0.515)	−0.0142 (0.054)	0.0062 (0.249)	−0.0081 (0.104)
Ln (Income) 50th percentile	−0.0086 * (0.097)	−0.0029 (0.427)	−0.0105 *** (0.000)	−0.0052 ** (0.012)
50th percentile * Treatment	0.0097 (0.144)	−0.0122 * (0.081)	0.0053 (0.223)	−0.0116 ** (0.014)
Ln (Income) 75th percentile	−0.0164 *** (0.003)	−0.0078 ** (0.042)	−0.0207 *** (0.000)	−0.0094 *** (0.001)
75th percentile * Treatment	0.0135 * (0.054)	−0.0183 ** (0.010)	0.0224 *** (0.000)	−0.0054 (0.226)
Proportion of AFHM	0.0675 *** (0.007)	0.0575 ** (0.011)	0.0555 *** (0.001)	0.0465 *** (0.002)
Constant	0.1386 *** (0.000)	0.1331 *** (0.000)	0.1455 *** (0.000)	0.1404 *** (0.000)
Observation	484	484	484	484
R-squared	0.1066	0.1047	0.1798	0.1963

Statistical significance: “’*” 10 percent, “**” 5 percent, “***” 1 percent. *p*-value in parentheses.

**Table 2:** The impacts of urban and rural hot (and cold) spots on obesity (2016).

Obesity	(1)	(2)	(3)	(4)
Treatment: Urban Hot Spots	−0.0278 *** (0.004)			
Treatment: Rural Hot Spots		0.0185 ** (0.077)		
Treatment: Urban Cold Spots			0.0034 (0.542)	
Treatment: Rural Cold Spots				0.0273 *** (0.000)
Ln(Income) 25th percentile	−0.0105 (0.275)	0.0022 (0.700)	−0.0087 *** (0.0076)	−0.0024 (0.596)
25th percentile * Treatment	0.0234 ** (0.035)	-0.0159 (0.216)	0.0004 (0.956)	−0.0248 *** (0.006)
Ln (Income) 50th percentile	-0.0013 (0.900)	-0.0123 (0.105)	−0.0100 *** (0.047)	−0.0003 ** (0.949)
50th percentile * Treatment	-0.0146 (0.227)	−0.0295 ** (0.022)	−0.0010 (0.886)	−0.0244 *** (0.008)
Ln (Income) 75th percentile	−0.0122 (0.189)	−0.0023 (0.662)	−0.0267 *** (0.000)	−0.0065 *** (0.230)
75th percentile * Treatment	0.0159 (0.129)	−0.0193 * (0.086)	0.0240 *** (0.002)	−0.0144 (0.113)
Proportion of AFHM	0.1680 *** (0.000)	0.02011 *** (0.000)	0.1126 *** (0.001)	0.0085 *** (0.001)
Constant	0.3489 *** (0.000)	0.3326 *** (0.000)	0.3549 *** (0.000)	0.3455 *** (0.000)
Observation	484	484	484	484
R-squared	0.2184	0.1676	0.1346	0.1802

Statistical significance: “*” 10 percent, “**” 5 percent, “***” 1 percent. *p*-value in parentheses.

## Appendix A. Figures and Tables

**Table A1:** Variable descriptive statistics for 2016 data (*t*-test in parentheses).

	Hotspot (Mean)	Non-Hotspot (Mean)	Difference	Coldspot (Mean)	Non-Coldspot (Mean)	Difference
Urban	0.6333	0.2149	−0.4183 (−10.0961)	0.3043	0.4821	0.1777 (3.1104)
Family Households (Proportion)	0.6149	0.6657	0.0508 (4.9497)	0.6608	0.6319	−0.0289 (−2.1837)
Unemployment (Proportion)	0.0404	0.4235	0.0019 (1.0623)	0.0388	0.0418	0.0029 (1.2688)
Health Insurance coverage (Proportion)	0.9040	0.8965	−0.0074 (−2.3575)	0.8965	0.9017	0.0052 (1.2973)
Food stamp/Snap Benefits (Proportion)	0.1588	0.1934	0.0345 (4.2315)	0.1767	0.1735	−0.0032 (−0.3095)
Mean Household Income (in 2016 dollars)	72,467.74	59,389.27	−13,078.47 (−7.2750)	60,906.92	68,041.22	7134.2 (3.0039)
Vehicle available	2350	2200	150 (−1.2173)	2180	2310	130 (0.8650)
Bachelor degree (Proportion)	0.1428	0.0861	−0.0566 (−10.8271)	0.0911	0.1240	0.0328 (4.5403)
Non-White (Proportion)	0.0757	0.0372	−0.0385 (−5.6177)	0.0327	0.0648	0.0321 (3.6340)
Age < 19 (Proportion)	0.1173	0.1116	−0.0056 (−1.4515)	0.1088	0.1162	0.0074 (1.5002)
Age > 59 (Proportion)	0.1694	0.1901	0.02075 (6.2027)	0.19425	0.1749	−0.0193 (−4.4825)
Community Health Services (Count)	0.2481	0.3878	0.1397 (3.3315)	0.4130	0.2857	−0.1273 (−2.3857)

**Table A2:** Covariate balancing.

	Treatment Mean (Hot Spots)	Control Mean (Before Weighting)	Control Mean (After Weighting)	Treatment Mean (Cold Spots)	Control Mean (Before Weighting)	Control Mean (After Weighting)
Proportion of Family Household	0.6149	0.6658	0.615	0.6609	0.6319	0.6609
Proportion of Unemployment	0.0404	0.0423	0.0404	0.0388	0.0418	0.0388
Proportion of Health Insurance Coverage	0.9041	0.8966	0.9041	0.8965	0.9018	0.8965
Proportion Food stamp/Snap benefits	0.1588	0.1934	0.1588	0.1768	0.1735	0.11768
Mean Household Income	72,468	59,389	72,468	60,907	68,041	60,907
Vehicle available	2351.6	2350.3	2351.6	2182.3	2311.2	2182.3
Proportion of Bachelor degree	0.1428	0.08616	0.1428	0.0911	0.124	0.09118
Proportion of Non-White	0.0757	0.0372	0.07576	0.0327	0.06484	0.03272
Proportion of age < 19	0.1173	0.1116	0.1173	0.1088	0.1162	0.1088
Proportion of age > 59	0.1694	0.1902	0.1694	0.1942	0.1749	0.1942
Community Health Services	0.2481	0.3879	0.2482	0.413	0.2857	0.413

**Table A3:** The impacts of hot and cold spots on diabetes rates.

Diabetes	(1) Unbalanced	(2) Balanced	(3) Unbalanced	(4) Balanced
Treatment: Hot Spots	−0.0041 *** (0.000)	−0.0054 *** (0.045)		
Treatment: Cold Spots			0.0052 *** (0.009)	0.0057 *** (0.002)
Constant	0.2236 *** (0.000)	0.1354 *** (0.000)	0.2190 *** (0.000)	0.1388 *** (0.000)
Observation	484	484	484	484
R-squared	0.3150	0.0216	0.2411	0.0322

Statistical significance: ‘*’10 percent, ‘**’ 5 percent, “***” 1 percent. *p*-value in parentheses.

**Table A4:** The impacts of hot and cold spots on obesity rates.

Obesity	(1) Unbalanced	(2) Balanced	(3) Unbalanced	(4) Balanced
Treatment: Hot Spots	−0.0128 *** (0.000)	−0.0188 *** (0.000)		
Treatment: Cold Spots			0.0052 *** (0.009)	0.0137 *** (0.002)
Constant	0.3639 *** (0.000)	0.3533 *** (0.000)	0.2190 *** (0.000)	0.3483 *** (0.000)
Observation	484	484	484	484
R-squared	0.3500	0.1162	0.3453	0.0660

Statistical significance: ‘*’10 percent, ‘**’ 5 percent, “***" 1 percent. *p*-value in parentheses.

**Table A5:** Propensity score weighting results for diabetes.

Diabetes	(1)	(2)	(3)	(4)
Treatment: Urban Hot Spots	−0.0116 *** (0.001)			
Treatment: Rural Hot Spots		0.0127 ** (0.015)		
Treatment: Urban Cold Spots			0.0151 (0.143)	
Treatment: Rural Cold Spots				0.0293 *** (0.008)
Ln(Income) 25th percentile	−0.0069 *** (0.040)	−0.0017 (0.511)	−0.0131 (0.332)	−0.0101 (0.500)
25th percentile * Treatment	0.0096 * (0.064)	−0.0099 (0.173)	0.0039 (0.783)	−0.0041 (0.793)
Ln (Income) 50th percentile	−0.0116 (0.002)	0.0055 * (0.075)	−0.0016 (0.892)	−0.0025 (0.847)
50th percentile * Treatment	−0.0131 ** (0.015)	-0.0109 (0.120)	−0.0047 (0.696)	−0.0218 (0.008)
Ln (Income) 75th percentile	−0.0200 *** (0.000)	−0.0114 *** (0.000)	−0.0218 * (0.057)	−0.0180 (0.134)
75th percentile * Treatment	0.0159 *** (0.004)	−0.0152 ** (0.027)	0.0261 *** (0.027)	−0.0046 (0.699)
Proportion of AFHM	0.0588 *** (0.003)	0.0540 *** (0.001)	0.2583 *** (0.000)	0.1702 *** (0.001)
Constant	0.1440 *** (0.000)	0.1380 *** (0.000)	0.1187 *** (0.000)	0.1167 *** (0.000)
Observation	484	484	484	484
R-squared	0.1440	0.1645	0.2881	0.3104

Statistical significance: “*” 10 percent, “**” 5 percent, “***” 1 percent. *p*-value in parentheses.

**Table A6:** Propensity score weighting results for obesity.

Obesity	(1)	(2)	(3)	(4)
Treatment: Urban Hot Spots	−0.0332 *** (0.000)			
Treatment: Rural Hot Spots		0.0117 (0.310)		
Treatment: Urban Cold Spots			0.0409 *** (0.000)	
Treatment: Rural Cold Spots				0.0523 *** (0.000)
Ln (Income) 25th percentile	−0.0132 ** (0.027)	−0.0015 (0.738)	−0.0052 (0.653)	−0.0014 (0.914)
25th percentile * Treatment	0.0290 *** (0.001)	−0.0112 (0.435)	0.0088 (0.532)	−0.0249 (0.137)
Ln (Income) 50th percentile	−0.0077 (0.248)	0.0059 (0.301)	−0.0059 (0.522)	−0.0131 (0.172)
50th percentile * Treatment	−0.0249 *** (0.009)	−0.0219 (0.108)	−0.0212 * (0.072)	−0.0376 (0.007)
Ln (Income) 75th percentile	−0.0174 ** (0.015)	−0.0081 *** (0.136)	−0.0165 * (0.053)	−0.0099 (0.194)
75th percentile * Treatment	0.0221 ** (0.013)	−0.0138 (0.272)	0.0186 (0.100)	−0.0047 (0.712)
Proportion of AFHM	0.1233 *** (0.003)	0.1548 *** (0.001)	0.4877 *** (0.000)	0.3388 *** (0.000)
Constant	0.3561 *** (0.000)	0.3411 *** (0.000)	0.3069 *** (0.000)	0.3033 *** (0.000)
Observation	484	484	484	484
R-squared	0.2311	0.1649	0.3931	0.4253

Statistical significance: “*” 10 percent, “**” 5 percent, “***” 1 percent. *p*-value in parentheses.

**Table A7:** Kernel matching results for diabetes.

Diabetes	(1)	(2)	(3)	(4)
Treatment: Urban Hot Spots	−0.0098 *** (0.008)			
Treatment: Rural Hot Spots		0.0129 ** (0.021)		
Treatment: Urban Cold Spots			0.0069 (0.404)	
Treatment: Rural Cold Spots				0.0146 *** (0.001)
Ln(Income) 25th percentile	−0.0049 (0.123)	−0.0007 (0.794)	−0.0053 * (0.093)	0.0006 (0.870)
25th percentile * Treatment	0.0068 (0.203)	−0.0111 (0.122)	0.0032 (0.745)	−0.0136 ** (0.029)
Ln (Income) 50th percentile	−0.0112 *** (0.000)	−0.0058 ** (0.016)	−0.0124 *** (0.000)	−0.0068 * (0.093)
50th percentile * Treatment	−0.0123 ** (0.014)	−0.0088 (0.202)	0.0072 (0.499)	−0.0105 * (0.098)
Ln (Income) 75th percentile	−0.0124 *** (0.000)	−0.0057 ** (0.019)	−0.0192 *** (0.000)	−0.0080 * (0.076)
75th percentile * Treatment	0.0095 ** (0.038)	−0.0194 *** (0.003)	0.0203 * (0.057)	−0.0084 (0.492)
Proportion of AFHM	0.0737 *** (0.000)	0.0728 *** (0.001)	0.0255 (0.252)	0.0136 (0.530)
Constant	0.1398 *** (0.000)	0.1344 *** (0.000)	0.1473 *** (0.000)	0.1412 *** (0.000)
Observation	484	484	484	484
R-squared	0.0876	0.0911	0.1311	0.1651

Statistical significance: “*” 10 percent, “**” 5 percent, “***” 1 percent. *p*-value in parentheses.

**Table A8:** Kernel matching results for obesity.

Obesity	(1)	(2)	(3)	(4)
Treatment: Urban Hot Spots	−0.0307 *** (0.000)			
Treatment: Rural Hot Spots		0.0187 ** (0.025)		
Treatment: Urban Cold Spots			0.0069 (0.619)	
Treatment: Rural Cold Spots				0.0383 *** (0.000)
Ln(Income) 25th percentile	−0.0131 *** (0.004)	−0.0113 (0.786)	−0.0017 (0.748)	0.0179 (0.003)
25th percentile * Treatment	0.0272 *** (0.000)	−0.0135 (0.206)	0.0095 (0.569)	−0.0413 ** (0.000)
Ln (Income) 50th percentile	−0.0008 (0.827)	−0.0058 ** (0.016)	−0.0042 (0.450)	0.0114 * (0.078)
50th percentile * Treatment	−0.0149 ** (0.039)	−0.0122 (0.001)	0.0067 (0.708)	−0.0363 *** (0.098)
Ln (Income) 75th percentile	−0.0185 *** (0.000)	−0.0080 ** (0.029)	−0.0175 *** (0.032)	−0.0048 (0.498)
75th percentile * Treatment	0.0234 *** (0.000)	−0.0165 * (0.092)	0.0140 (0.431)	−0.0280 (0.152)
Proportion of AFHM	0.127 *** (0.000)	0.1518 *** (0.000)	0.0720 * (0.055)	0.0386 (0.530)
Constant	0.3524 *** (0.000)	0.3339 *** (0.000)	0.3527 *** (0.000)	0.3387 *** (0.000)
Observation	484	484	484	484
R-squared	0.2154	0.1649	0.0611	0.1859

Statistical significance: “*” 10 percent, “**” 5 percent, “***” 1 percent. *p*-value in parentheses.

**Table A9:** The impacts of urban and rural hot (and cold) spots on diabetes (2017).

Diabetes	(1)	(2)	(3)	(4)
Treatment: Urban Hot Spots	−0.0087 ** (0.050)			
Treatment: Rural Hot Spots		0.0137 ** (0.018)		
Treatment: Urban Cold Spots			−0.0069 ** (0.031)	
Treatment: Rural Cold Spots				0.0127 *** (0.001)
Ln(Income) 25th percentile	0.0008 (0.848)	0.0009 (0.782)	−0.0076 *** (0.002)	−0.0030 (0.143)
25th percentile * Treatment	0.0001 (0.977)	−0.0025 (0.735)	0.0043 (0.519)	−0.0098 * (0.058)
Ln (Income) 50th percentile	−0.0086 * (0.085)	−0.0033 (0.445)	−0.0108 *** (0.000)	−0.0055 * (0.009)
50th percentile * Treatment	−0.0144 ** (0.025)	−0.0102 (0.164)	0.0083 ** (0.030)	−0.0092 * (0.087)
Ln (Income) 75th percentile	−0.0179 *** (0.001)	−0.0086 * (0.052)	−0.0162 *** (0.000)	−0.0094 *** (0.001)
75th percentile * Treatment	0.0170 *** (0.013)	−0.0164 ** (0.026)	0.0204 *** (0.000)	−0.0054 (0.348)
Proportion of AFHM	0.0840 *** (0.001)	0.0753 *** (0.001)	0.0580 *** (0.000)	0.0478 *** (0.001)
Constant	0.1371 *** (0.000)	0.1327 *** (0.000)	0.1454 *** (0.000)	0.1402 *** (0.000)
Observation	484	484	484	484
R-squared	0.1462	0.1255	0.1646	0.1905

Statistical significance: “*” 10 percent, “**” 5 percent, “***” 1 percent. *p*-value in parentheses.

**Table A10:** The impacts of urban and rural hot (and cold) spots on obesity (2017).

Obesity	(1)	(2)	(3)	(4)
Treatment: Urban Hot Spots	−0.0260 *** (0.001)			
Treatment: Rural Hot Spots		0.0212 ** (0.049)		
Treatment: Urban Cold Spots			−0.0004 (0.946)	
Treatment: Rural Cold Spots				0.0284 *** (0.001)
Ln(Income) 25th percentile	0.0005 (0.948)	0.0036 (0.519)	−0.0090 * (0.084)	−0.0032 (0.485)
25th percentile * Treatment	0.0100 (0.302)	−0.0100 (0.458)	0.0029 (0.742)	−0.0297 *** (0.002)
Ln (Income) 50th percentile	−0.0044 (0.652)	−0.0163 ** (0.043)	−0.0107 ** (0.040)	0.00008 (0.985)
50th percentile * Treatment	−0.0139 (0.222)	−0.0343 ** (0.010)	0.0034 (0.639)	−0.0232 ** (0.018)
Ln (Income) 75th percentile	−0.0117 (0.174)	−0.0024 (0.671)	−0.0190 *** (0.002)	−0.0092 * (0.089)
75th percentile*Treatment	0.0186 * (0.059)	−0.0234 ** (0.045)	0.0240 *** (0.003)	−0.0049 (0.642)
Proportion of AFHM	0.1650 *** (0.000)	0.2053 *** (0.000)	0.1215 *** (0.000)	0.0478 *** (0.001)
Constant	0.3453 *** (0.000)	0.3314 *** (0.000)	0.3544 *** (0.000)	0.3449 *** (0.000)
Observation	484	484	484	484
R-squared	0.2515	0.2131	0.1266	0.2003

Statistical significance: “*” 10 percent, “**” 5 percent, “***” 1 percent. *p*-value in parentheses.
